# The Histone Deacetylase Gene *Rpd3* Is Required for Starvation Stress Resistance

**DOI:** 10.1371/journal.pone.0167554

**Published:** 2016-12-01

**Authors:** Ei Nakajima, Kouhei Shimaji, Takanari Umegawachi, Saki Tomida, Hideki Yoshida, Nana Yoshimoto, Shingo Izawa, Hiroshi Kimura, Masamitsu Yamaguchi

**Affiliations:** 1 Department of Applied Biology, Kyoto Institute of Technology, Kyoto, Japan; 2 Insect Biomedical Research Center, Kyoto Institute of Technology, Kyoto Japan; 3 Graduate School of Bioscience and Biotechnology, Tokyo Institute of Technology, Yokohama, Japan; Biomedical Sciences Research Center Alexander Fleming, GREECE

## Abstract

Epigenetic regulation in starvation is important but not fully understood yet. Here we identified the *Rpd3* gene, a *Drosophila* homolog of *histone deacetylase 1*, as a critical epigenetic regulator for acquiring starvation stress resistance. Immunostaining analyses of *Drosophila* fat body revealed that the subcellular localization and levels of Rpd3 dynamically changed responding to starvation stress. In response to starvation stress, the level of Rpd3 rapidly increased, and it accumulated in the nucleolus in what appeared to be foci. These observations suggest that Rpd3 plays a role in regulation of rRNA synthesis in the nucleolus. The RT-qPCR and ChIP-qPCR analyses clarified that Rpd3 binds to the genomic region containing the rRNA promoters and activates rRNA synthesis in response to starvation stress. Polysome analyses revealed that the amount of polysomes was decreased in *Rpd3* knockdown flies under starvation stress compared with the control flies. Since the autophagy-related proteins are known to be starvation stress tolerance proteins, we examined autophagy activity, and it was reduced in *Rpd3* knockdown flies. Taken together, we conclude that Rpd3 accumulates in the nucleolus in the early stage of starvation, upregulates rRNA synthesis, maintains the polysome amount for translation, and finally increases stress tolerance proteins, such as autophagy-related proteins, to acquire starvation stress resistance.

## Introduction

Since the natural environment is constantly changing, all organisms have to adapt to the changing environment and acquire tolerance to various types of stress. A number of studies have revealed the possible mechanisms for this adaptation to oxidation [1] and temperature [2] stresses. These studies have mostly focused on identifying signaling pathway(s) responding to these stresses. The basic pathways responding to stress appear to be shared regardless of the type of stress. Under stress, first, a sensor senses the environmental changes and transmits signals into the cells. The MAP-kinase (MAPK) signaling pathway is well known as a commonly important pathway in the stress response [3] and is highly conserved from yeast to human [4]. In mammalian cells, p38 and c-Jun N-terminal kinases (JNK) are representative stress-responding MAPKs. In general, phosphorylation of several transcription factors by the activated MAPKs causes the increased expression of stress tolerance genes. Although the forkhead box O (FOXO) transcription factor and target of rapamycin complexes (TORCs) are well known components of the responding pathway to starvation stress, their detailed regulatory mechanisms remain poorly understood [5]. Furthermore, most of previous studies mainly focused on the signaling cascades related to stress responses, and transcriptional regulation of genes involved in these cascades have not been fully studied yet.

Recently, a number of studies have indicated that epigenetic regulation plays an important role in the oxidative stress response by regulating the expression of several stress tolerance genes and also the genes encoding the signaling cascade proteins [6,7]. However, it is still not yet fully clarified which epigenetic regulator(s) plays a critical role in starvation stress, except for Sirtuin 2, a histone deacetylase (HDAC) that is suggested to play a role in starvation stress resistance in yeast [8]. Moreover, most of the studies on epigenetics related to starvation stress have been performed with a unicellular organism like yeast as a model [9–11]. In the present study, we tried to clarify the mechanism of epigenetic regulation to acquired tolerance for starvation stress by using *Drosophila melanogaster* as a model organism, since *Drosophila* has already been established as an excellent model for studying the genetic control of metabolic pathways, sharing most of the basic metabolic pathways with human [5]. The similarity between *Drosophila* and human even extends our knowledge on the progression of metabolic diseases caused by dysfunctions in metabolism [12]. We have identified the histone deacetylase gene *Rpd3* as the most critical gene in starvation stress tolerance in *Drosophila*. We also performed the functional analyses of *Rpd3* and revealed the role of *Rpd3* in acquiring tolerance to starvation stress.

## Results

### *Rpd3* knockdown flies die much faster than the control in starvation

**Fig 1:**
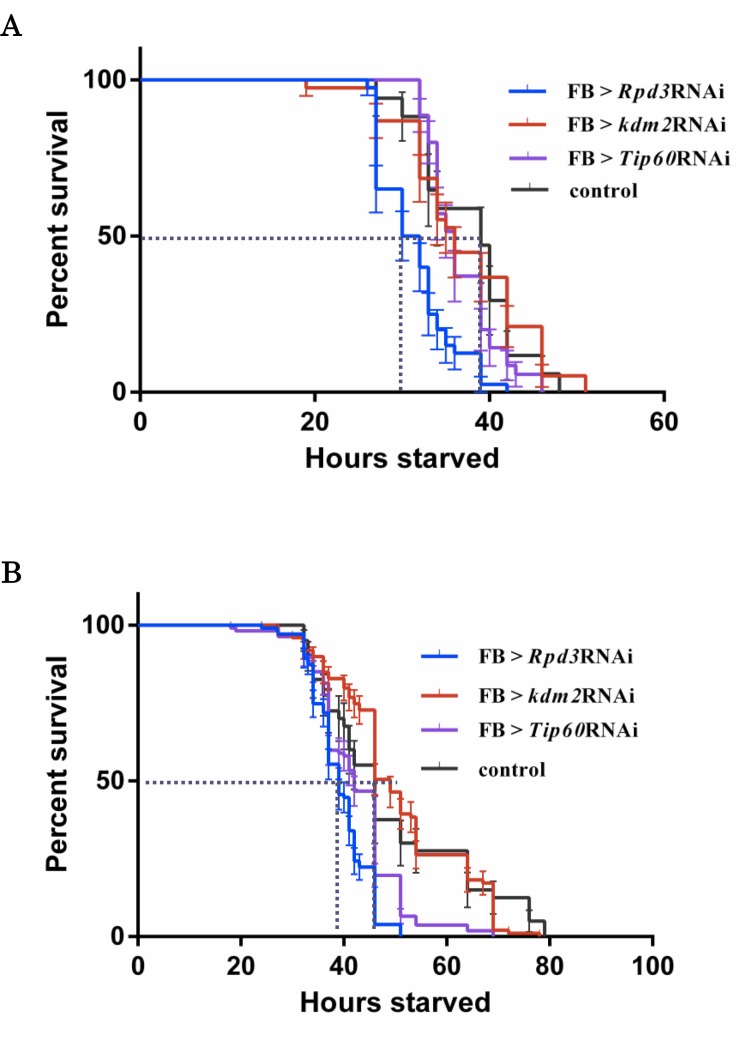
Viability of RNAi lines for several epigenetic regulators under starvation stress. **A**. Percent survival of adult male flies under starvation. **B.** Percent survival of adult female flies under starvation. FB > *Rpd3*RNAi (*w*; *Fb-GAL4* /+; *HDAC1*^*JF01401*^/+), FB > *kdm2*RNAi (*w*; *Fb-GAL4* / +; *Kdm2*^*HMS00574*^ /+), FB > *Tip60*RNAi (*w*; *Fb-GAL4* / +; *Tip60*^*GL00130*^ /+), control (*w*; *Fb-GAL4* / +). Male and female *Rpd3* knockdown flies’ starvation half-life was 30 h and 39 h, respectively. Male and female control flies’ starvation half-life was 39 h and 46 h, respectively.

Genome-wide microarray analyses of *Drosophila* mRNAs under starvation half-life conditions have been reported [13]. In the report, 3,451 probe sets with significantly different mean transcript levels between the control and starved conditions were found [13]. 1,715 probes are upregulated, and 1,736 probes are down-regulated [13]. Based on these data, we performed starvation assays with RNAi lines and mutants for the candidate genes in order to determine which epigenetic regulators are important to overcome starvation stress. We focused on genes carrying the following two criteria for identification of candidate epigenetic regulators. First, we selected the genes whose changes in expression levels under starvation are statistically significant (P < 0.001). Second, we selected genes that are well-known as epigenetic regulators. Six genes, *HDAC4*, *Rpd3*, *CG2051*, *EG*:*EG00077* (*Tip60*), *Mes-1*, and *Ash* satisfy these criteria. According to the microarray analyses, the gene expression levels are significantly changed in these genes under starvation (fold-change; *HDAC4*: 0.555, *Rpd3*: 1.303, *CG2051*: 1.397, *Tip60*: 1.447, *Mes-1*: 1.653, *Ash*: 1.517). *HDAC4* and *Rpd3* are known as histone deacetylase genes. *CG2051* and *EG*:*EG00077* (*Tip60*) are known as histone acetyltransferase genes. *Mes-1* and *Ash* are known as histone methylase genes. The fat body (FB) in *Drosophila* is known for its importance in not only energy storage but also lipid metabolism, similar to the mammalian liver [14]. It is reported that *Drosophila* fat body plays an essential role in systemic metabolic homeostasis under fasting conditions [15–17]. We therefore performed starvation assays with fat body-specific knockdown flies for the candidate genes such as *Rpd3* and *Tip60*. RNAi lines for the non-candidate epigenetic regulator *kdm2* (histone H3K4–specific methylase) were also tested as a negative control (Fig 1A and 1B).

The male starvation half-life of *Rpd3* knockdown flies was 30 h, that of *Tip60* knockdown flies was 36 h, that of *kdm2* knockdown flies was 42 h, and that of the control flies was 39 h (Fig 1A). These results indicate that *Rpd3* knockdown flies are the most sensitive to starvation stress among the examined candidates. Similarly, the female starvation half-life of *Rpd3* knockdown flies was 39 h, that of *Tip60* knockdown flies was 42 h, and those of *kdm2* knockdown flies and control flies were 46 h (Fig 1B). In these starvation assays, *Rpd3* knockdown flies showed the highest sensitivity to starvation stress regardless of gender. Furthermore, previous studies in yeast and human cells suggested that regulation of the histone acetylation state of the promoter region is important for transcriptional regulation of the stress tolerance genes [18]. We therefore performed more detailed analyses with the male *Rpd3* knockdown flies.

In order to exclude possible off-target effects, we performed starvation assays with two independent *Rpd3* knockdown flies designed for different target regions in *Rpd3* mRNA (S1 Fig). The starvation half-life of the FB-GAL4>UAS-*Rpd3*IR (FB-GAL4; *Rpd3*^*JF01401*^/+) flies was 24 h, and that of the FB-GAL4>UAS-*Rpd3*IR (FB-GAL4; *Rpd3*^*GL01005*^/+) flies was 30 h, while that of the control flies was 36 h. These results indicate that the reduced starvation half-life of *Rpd3* knockdown flies is not due to off-target effects but due to the reduced level of Rpd3. Thereafter, we used the former FB-GAL4>UAS-*Rpd3*IR lines for the detailed analyses. Additionally, we performed the Western immunoblot analysis with anti-Rpd3 antibody (S2A Fig). Two bands were detected; the upper band corresponds to 93.8 kDa, and the lower band to 67.7 kDa. The signal intensities of these two bands relative to α-tubulin were quantified (S2B and S2C Fig). In *Rpd3* knockdown flies, at 6 h starvation the upper band and lower band decreased to 44% and 49%, respectively, compared with the wild type flies, indicating that both bands are related to Rpd3. The molecular mass of *Drosophila* Rpd3 based on its amino acid composition is 63 kDa. Therefore, the lower 67.7 kDa band very likely represents Rpd3. The upper 93.8 kDa band may represent some modified form of Rpd3, such as a phosphorylated one or some undescribed splicing variant. In yeast, a similar higher molecular weight form of Rpd3 was also described, although no further analysis was performed [19]. In order to evaluate the knockdown efficiency of *Tip60*, the gene expression analysis was carried out with RT-qPCR by measuring the relative level of *Tip60* transcripts, with respect to *Glucose-6-phosphate dehydrogenase* (*G6pd*) mRNA as an internal control (S2D Fig). Flies carrying FB-GAL4>UAS-*Tip60*IR exhibited 49% reduction in *Tip60* mRNA levels. In any event, these data confirmed that Rpd3 and Tip60 is efficiently knocked down in the FB-GAL4>UAS-*Rpd3*IR (FB-GAL4; *Rpd3*^*JF01401*^/+) and FB-GAL4>UAS-*Tip60*IR (FB-GAL4; *Tip60*^*GL00130*^ /+) flies.

We also performed the starvation assay with *Rpd3* overexpression flies (S1 Fig). *Rpd3* overexpression flies showed a starvation half-life of 27 h that was significantly shorter than that of the control flies, indicating that *Rpd3* overexpression flies are also sensitive to starvation stress. These results suggest that proper regulation of *Rpd3* expression under starvation stress is important for starvation stress tolerance.

### Rpd3 signals form foci in the nucleolus, and the number of foci increases in response to starvation stress

**Fig 2:**
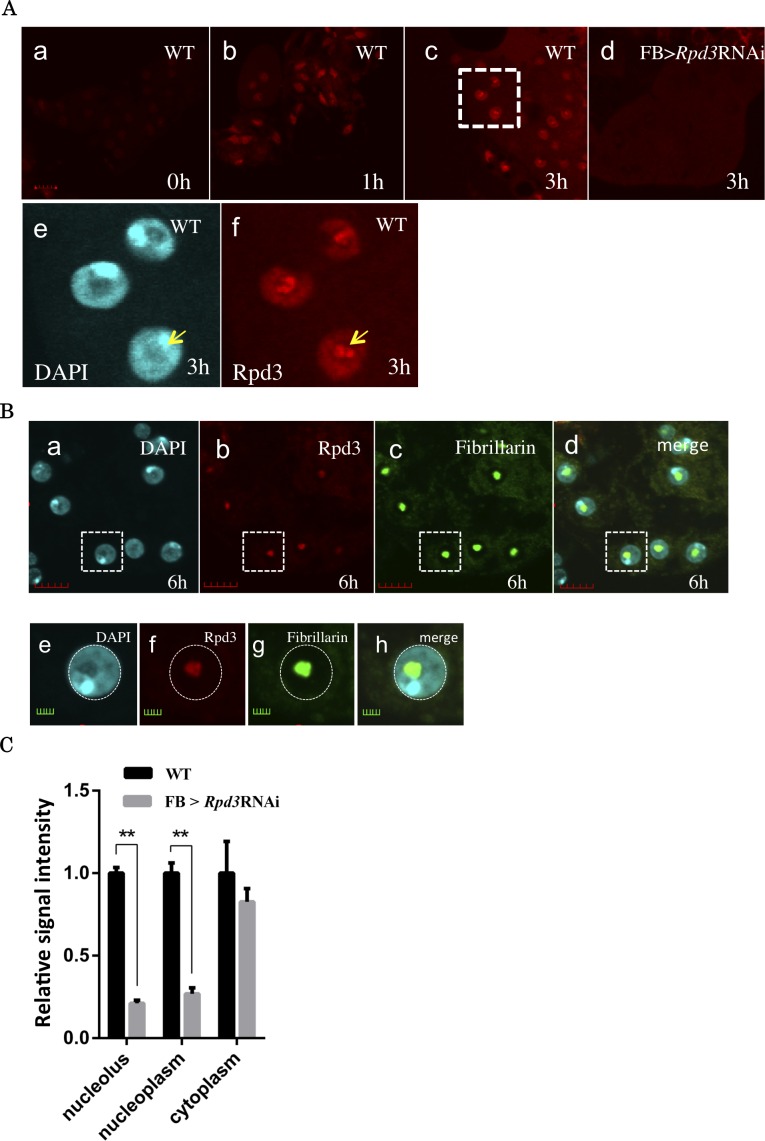
Rpd3 signals form foci in the nucleolus, and the number of foci increases in response to starvation stress. **A.** Sub-cellular localization of Rpd3 in *Drosophila* adult fat bodies. (a to d and f) Immunostaining with anti-Rpd3 IgG. (e) DAPI staining. (a to c) Wild type Canton S flies starved for 0 h (a), 1 h (b) and 3 h (c). (d) *Rpd3* knockdown flies (*w*; *Fb-GAL4* /+; *HDAC1*^*JF01401*^/+) starved for 3 h. (e and f) Enlarged image of the region shown in panel c. Scale bars: (a-d) 10 μm, (e-f) 5 μm. **B.** Sub-cellular localization of Rpd3 in 6 h starved wild type *Drosophila* adult fat bodies. (a) DAPI staining. (b) Immunostaining with anti-Rpd3 IgG. (c) Immunostaining with anti-Fibrillarin IgG (d) Merged image of (a) to (c). (e to h) Enlarged images of the indicated regions in (a to d). Rpd3 signals co-localized with Fibrillarin (nucleolus marker). Scale bars: (a-d) 5 μm, (e-h) 2 μm. **C.** Comparison of the Rpd3 signal strength between wild type Canton S (WT) and *Rpd3* knockdown flies (*w*; *Fb-GAL4* /+; *HDAC1*^*JF01401*^/+) at 3 h starved. Rpd3 signals are significantly stronger in the nucleolus and nucleoplasm. ** P-value < 0.01. n = 7.

The previous microarray analyses revealed that *Rpd3* mRNA increased to 1.3 fold under starvation half-life conditions [13]. However, sub-cellular localization of Rpd3 and its dynamics under starvation are not known yet. First, we performed immunostaining of the fat body with anti-Rpd3 antibody (Fig 2). The fat body in *Drosophila* plays an important role in acquiring tolerance under starvation stress [14–17]. We observed the Rpd3 signal intensity increase from 0 h to 3 h in wild type *Drosophila* fat bodies (Fig 2A a-c). At 1 h starvation, the Rpd3 signal was detected mainly in the nucleoplasm and also in the cytoplasm at low levels (Fig 2Ab). Then, the Rpd3 signal started to form foci at 3 h starvation (Fig 2Ac, e, f). The foci were observed up until 6 h starvation. To determine where these foci were located, we next performed double-immunostaining analyses with anti-Rpd3 antibody in combination with the nucleolus marker, anti-Fibrillarin antibody (Fig 2B). The strong Rpd3 signals clearly merged with the Fibrillarin signals, indicating that Rpd3 mainly localizes in the nucleolus. These results indicate that Rpd3 starts to form foci in the nucleolus in response to starvation stress (0–6 h after onset of starvation). Only a low level of Rpd3 signal was detected in the fat body of *Rpd3* knockdown flies, also confirming the effective knockdown (Fig 2A-d). The quantified data of Rpd3 signals further confirmed the reduction of the Rpd3 signal in both the nucleolus and nucleoplasm, but not in the cytoplasm in the *Rpd3* knockdown flies (Fig 2C).

### Rpd3 binds to the genomic region containing the rRNA promoter and activates rRNA synthesis

**Fig 3:**
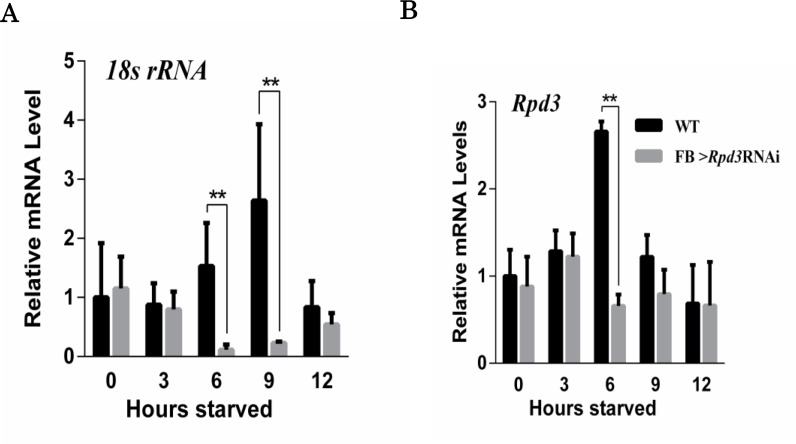
Transient induction of 18s rRNA and *Rpd3* mRNA in response to starvation. **A.** Relative 18s rRNA levels during starvation. 18s rRNA levels in adult male flies increased at 6 h and 9 h starved in Canton S (WT), but not in *Rpd3* knockdown flies (*w*; *Fb-GAL4* /+; *HDAC1*^*JF01401*^/+). **B.** Relative *Rpd3* mRNA levels during starvation. *Rpd3* mRNA levels in adult male flies increased at 6 h and 9 h starved in Canton S (WT), but not in *Rpd3* knockdown flies (*w*; *Fb-GAL4* /+; *HDAC1*^*JF01401*^/+). ** P-value < 0.01, n = 3.

Since it is well known that the nucleolus is the site of rRNA synthesis and processing, the observed extensive Rpd3 localization in the nucleolus under starvation (Fig 2B) suggests that Rpd3 plays a role in the regulation of rRNA synthesis. To address this possibility, first we quantified levels of *18s rRNA* under starvation by RT-qPCR analyses (Fig 3A). Levels of *18s rRNA* increased at 6 h and 9 h starvation in wild type flies, but not in *Rpd3* knockdown flies. At 12 h starvation, *18s rRNA* levels decreased in the wild type to levels similar to those in the *Rpd3* knockdown flies. Interestingly, *Rpd3* mRNA levels also increased at 6 h starvation, and this increase was not observed in the *Rpd3* knockdown flies (Fig 3B). Since a fat body-specific *Rpd3* knockdown fly was used for the RT-qPCR analyses, the abundance of *Rpd3* mRNA from other tissues likely masks the reduction of *Rpd3* mRNA at time points other than 6 h starvation. These results strongly suggest that Rpd3 regulates rRNA synthesis under starvation stress.

**Fig 4:**
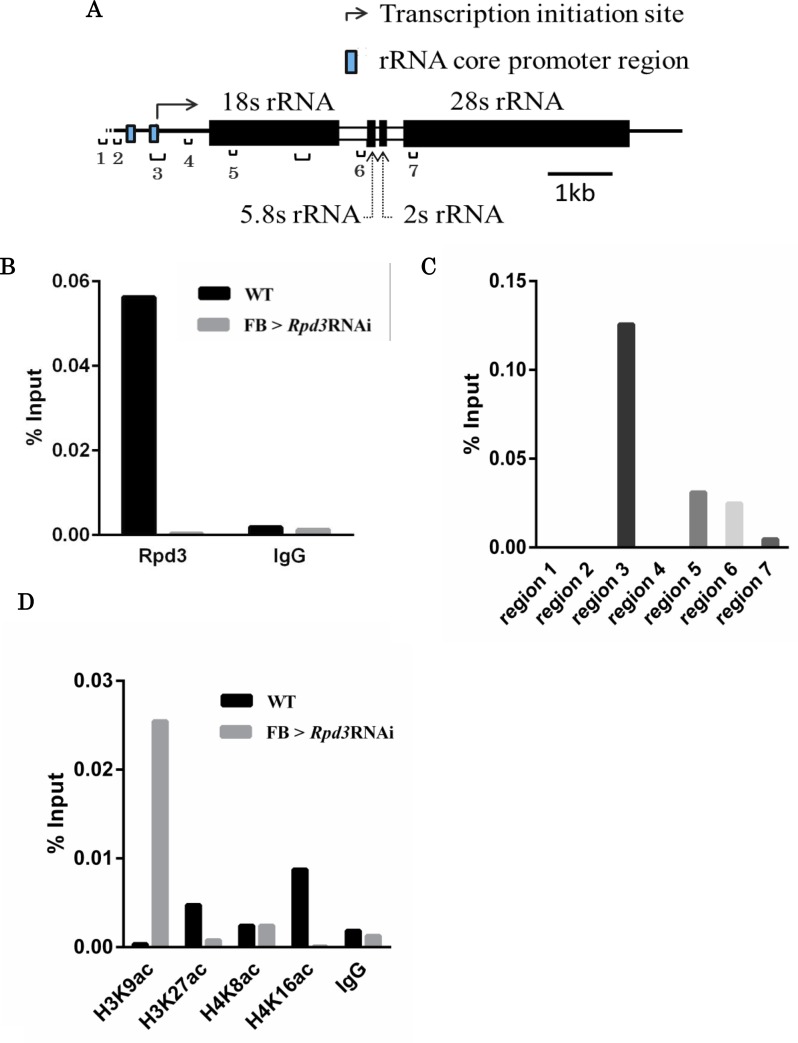
Rpd3 binds to the genomic region containing the rRNA core promoter. **A.** Schematic illustrations of the *Drosophila rRNA* gene region. The amplified regions 1 to 7 in the ChIP-qPCR assay are shown. Region 3 corresponds to the rRNA core promoter region. The amplified region in the RT-qPCR is also shown as the bracket without numbers. **B.** Rpd3 binds to the *18srRNA* promoter region in wild type flies but not in the *Rpd3* knockdown flies. CHIP–qPCR assays were performed with Canton S (WT) or *Rpd3* knockdown (*w*; *Fb-GAL4* /+; *HDAC1*^*JF01401*^/+) adult male flies at 6 h starved. Either anti-Rpd3 IgG or control IgG was used for the chromatin immunoprecipitation. Region 3, corresponding to the rRNA core promoter region, was amplified. **C**. Rpd3 binds specifically to the *18s rRNA* core promoter region. ChIP–qPCR assays were performed with Canton S adult male flies at 6 h starved. The primer sets for qPCR were designed for regions 1 to 7, shown in panel A. **D**. Acetylation state of histones H3 and H4 under starvation. ChIP–qPCR assays were performed with Canton S (WT) or *Rpd3* knockdown (*w*; *Fb-GAL4* /+; *HDAC1*^*JF01401*^/+) adult male flies at 6 h starved. Either anti-histone H3K9ac IgG (H3K9ac), anti- histone H3K27ac IgG (H3K27ac), anti-histone H4K8ac IgG (H4K8ac), anti-histone H4K16ac IgG (H4K16ac), or normal mouse IgG (IgG) was used for the chromatin immunoprecipitation. The primer sets for qPCR were designed for region 3, corresponding to the rRNA core promoter region.

We next performed ChIP-qPCR analyses of adult male flies with anti-Rpd3 antibody to examine Rpd3-binding to the genomic region containing the core rRNA promoter region in wild type and *Rpd3* knockdown flies (Fig 4B). The genomic region containing the rRNA promoter (region 3 in Fig 4A) was extensively amplified in the chromatin-immunoprecipitates from the wild type flies, but not from the *Rpd3* knockdown flies (Fig 4B). However, in the wild type flies, no amplification was observed in regions 1, 2, and 4, and only a marginal amplification was detected in the downstream regions 5, 6, and 7 (Fig 4A and 4C). These results revealed that Rpd3 binds specifically to the genomic region containing the rRNA promoters.

To obtain further insight into the mechanism of Rpd3 function in the regulation of rRNA synthesis in response to starvation, we analyzed the acetylation state of histones in the genomic region containing the rRNA core promoter by performing ChIP-qPCR with several acetylated histone antibodies, such as anti-H3K9ac, anti-H3K27ac, anti-H4K8ac, and anti-H4K16ac. H3K9 acetylation and H3K27 acetylation are well known as the active transcription feature of chromatin [20,21]. Acetylation of H3K9 and H4K16 has been reported to be catalyzed by Rpd3 in yeast [22]. Additionally, H4K16 acetylation is known to play a critical role in the regulation of higher order chromatin structure [23]. Although detailed analyses on H4K8 acetylation have not been carried out yet, it is predicted to be involved in transcriptionally-active chromatin formation [24].

The acetylated H3K9 level in the rRNA promoter region was low in the wild type flies and high in the *Rpd3* knockdown flies, suggesting that Rpd3 is involved in the regulation of acetylation levels of histone H3K9 in this genomic region (Fig 4D). The acetylated H4K8 level in the rRNA promoter region of the wild type flies was almost the same with that of the *Rpd3* knockdown flies, indicating that Rpd3 is not involved in the regulation of acetylation levels of H4K8 in this genomic region (Fig 4D). The acetylated H3K27 and H4K16 levels in the rRNA promoter region of the wild type flies were higher than those of the *Rpd3* knockdown flies, indicating also that Rpd3 is involved in the regulation of acetylation levels of H3K27 and H4K16 in this genomic region (Fig 4D). Previous studies in yeast showed that the acetylation of the histone H4K16 residue is involved in upregulation of rRNA synthesis [25]. Therefore, the hyper-acetylation of the H4K16 residue in this genomic region may play a major role in upregulation of 18s rRNA synthesis in response to starvation (Fig 3A).

### *Rpd3* knockdown reduced the amount of polysomes

**Fig 5:**
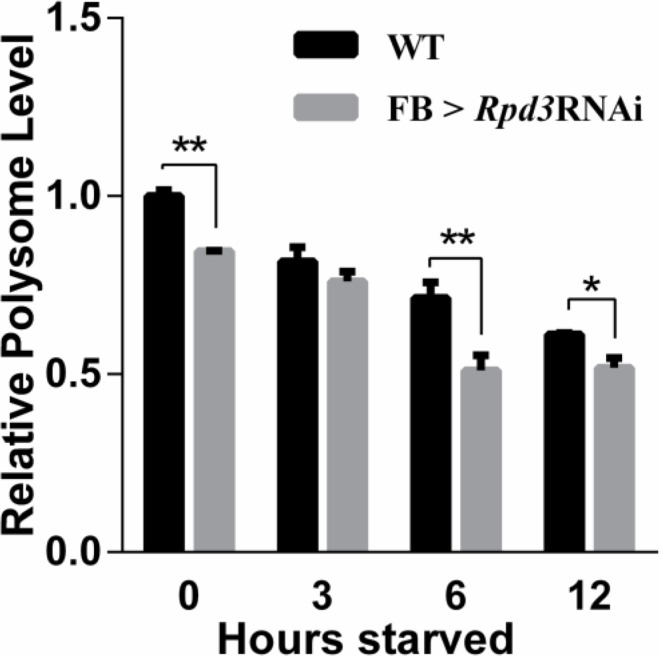
Knockdown of *Rpd3* reduces the amount of polysomes. Polysome profile analyses were performed with Canton S (WT) or *Rpd3* knockdown (*w*; *Fb-GAL4* /+; *HDAC1*^*JF01401*^/+) adult male flies at indicated hours starved. *Rpd3* knockdown flies showed less polysome amount than that of wild type flies, at 6 h and 12 h starvation. *P-value < 0.05 and ** P-value < 0.01, n = 2.

We next performed polysome-profiling analyses to evaluate translational activity under short-term starvation in both wild type and *Rpd3* knockdown flies. In wild type flies, the amount of polysomes gradually and progressively reduced under starvation (Fig 5). These observations were consistent with the previous studies with yeast and mammalian cells, indicating that generally global protein synthesis rates decrease under starvation stress [26]. As expected, in *Rpd3* knockdown flies, the amount of polysomes more rapidly reduced, responding quickly to starvation stress that was most prominent at 6 h starvation (Fig 5).

### *Rpd3* knockdown reduced mRNA levels of *Atg9*, a marker of autophagy activity

**Fig 6:**
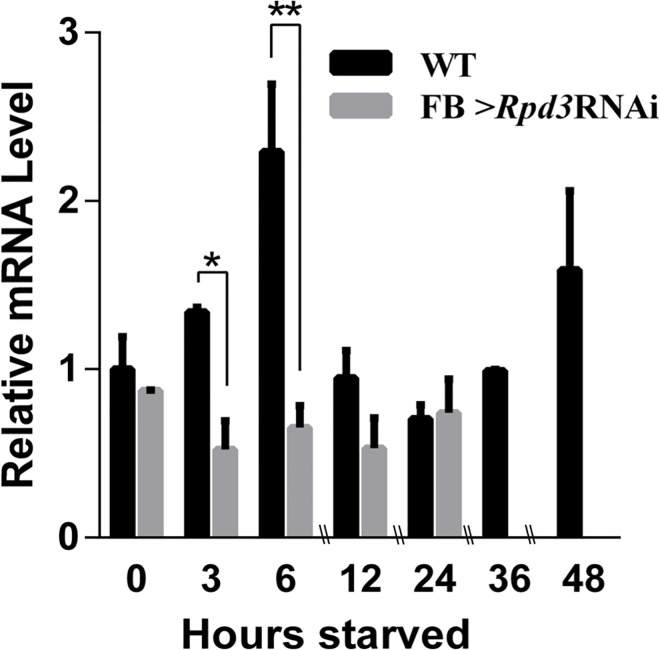
Knockdown of *Rpd3* reduces the level of *Atg9* mRNA that is induced in response to starvation. Relative *Atg9* mRNA levels were measured by RT-qPCR with Canton S (WT) or *Rpd3* knockdown (*w*; *Fb-GAL4* /+; *HDAC1*^*JF01401*^/+) adult male flies at indicated hours starved. The *Atg9* mRNA levels transiently increased at 3 h and 6 h starvation in wild type flies, but not in the *Rpd3* knockdown flies. Since *Rpd3* knockdown flies die at 36 h and 48 h starvation, data at these time points were not obtained with these flies. *P-value < 0.05 and ** P-value < 0.01, n = 3.

It has been reported that in spite of the decrease in global protein synthesis, mRNAs of stress resistance genes are actively translated under starvation [27]. Induction of autophagy is well-known to be a key cellular response to starvation stress. In this process, the autophagy-related genes are upregulated [28] and one of these genes, *Atg9* is commonly used to monitor autophagy activation [29]. We therefore next analyzed the expression pattern of *Atg9* mRNA under starvation by RT-qPCR analyses (Fig 6). In the wild type, *Atg9* mRNA quickly increased at 3 h and 6 h starvation, but these increases did not occur in *Rpd3* knockdown flies, indicating that autophagy-related proteins were not induced in *Rpd3* knockdown flies under starvation (Fig 6). We also performed ChIP-qPCR with anti-Rpd3 antibody to examine the possible binding of Rpd3 to the genomic region containing the *Atg9* promoter. However, no indication of Rpd3-binding to this genomic region was observed (data not shown). Taken together, these results suggest that the reduction of autophagy activity was not due to direct transcriptional regulation by Rpd3-binding, but mediated by translational regulation of autophagy-related proteins. The reduction of autophagy activity in *Rpd3* knockdown flies could explain why *Rpd3* knockdown flies died much faster than wild type flies under starvation stress.

## Discussion

In this study, by using *Drosophila* as a model system, we were able to show that epigenetic regulation is important to acquire tolerance to starvation stress, and Rpd3 plays a major role in this process. Here, we showed the striking nucleolus localization of Rpd3 under starvation stress. ChIP-qPCR analyses indicated that Rpd3 binds to the genomic region containing the promoter region of the rRNA gene, and histone H4K16 is hyper-acetylated in this genomic region. These results directly link the role of Rpd3 to activation of rRNA synthesis, since the acetylation of the H4K16 residue has been demonstrated to be involved in upregulation of rRNA transcription [25]. Previous studies in yeast reported that TOR negatively regulates association of Rpd3 with rDNA chromatin to promote rRNA gene transcription [30]. Rapamycin and nutrient starvation inhibit TOR, leading to nucleolar size reduction, accompanied by the release of RNA polymerase I from the nucleolus to inhibit rRNA synthesis that appears to be mediated by Rpd3 [30]. Later studies in yeast demonstrated that rRNA genes can be transcribed by RNA polymerase II in addition to RNA polymerase I, although RNA polymerase II transcription is usually silenced [31]. Rpd3 is suggested to be responsible for this polymerase switch by maintaining the rRNA gene chromatin structure [31]. In addition, in contrast to the previous report, Rpd3 is not required for inhibition of RNA polymerase I transcription by rapamycin [31]. Although the situation is rather complicated, at least yeast Rpd3 affects the rRNA gene chromatin structure and RNA polymerase I localization and transcription of rRNA genes.

In the present study, we suggest that Rpd3 upregulates H4K16 acetylation, even though this enzyme is well known as a histone deacetylase. Other previous studies also reported these similarly contradictory results. In mouse ES cells, HDAC1, the human homolog of Rpd3, directly upregulates histone acetylation and works as a positive regulator of transcription of some specific genes, like interferon (IFN) target genes [32]. Moreover, in yeast cells, disruption of the *Rpd3* gene deacetylates histones and retards *PHO5* gene promoter activation [33]. Therefore, these apparently contradictory acetylation states of histones may be explained by the balance of the antagonistic activities of histone acetyltransferases and histone deacetylases *in vivo*.

The immunostaining data indicate that Rpd3 signals increase not only in the nucleolus but also in the nucleoplasm under starvation stress (Fig 2). Although the role of Rpd3 located in the nucleoplasm is still unclear, it may relate to the regulation of genes involved in a well-known stress responding signaling pathway, such as the mTOR pathway [34]. Further analyses are necessary to clarify this point. Additionally, it would also be interesting to clarify the mechanisms of nucleolus localization of Rpd3 since such mechanisms, with the exception of ribosomal proteins, have not been studied yet.

In the ChIP-qPCR analyses with several histone acetylation antibodies, a striking increase in the acetylation level of H3K9 at the rRNA promoter locus was observed in the *Rpd3* knockdown flies, although this does not correlate with activation of rRNA transcription. Generally, histone H3 acetylation, especially at K9, is correlated with transcriptionally active euchromatin regions [20]. However, this appears to not be the case with the rRNA gene locus. Differences in transcriptional machinery between rRNA genes and other nuclear genes may be responsible for this difference.

Cells inside of tumor masses are under starved conditions, since nutrients are not fully supplied to these cells [35,36]. In order to overcome starvation stress, autophagy is induced in these cells [35,36]. It is well known that HDAC1, a human homolog of Rpd3, is highly expressed in hepatocellular carcinoma. HDAC inhibitors are now being tested for cancer therapy, since it can arrest the cell cycle and activate the extrinsic and intrinsic pathways of apoptosis [37,38]. However, the mechanisms by which HDAC1 is effective in preventing tumor formation is still unclear. The present study strongly suggests that Rpd3 is required for starvation resistance that is favorable for tumor mass formation. Inhibition of Rpd3 activity could induce cell death of starved cells inside the tumor mass. This may at least partially explain the working mechanism of HDAC inhibitors in cancer therapy.

## Materials and Methods

### Fly stocks

Fly stocks were cultured at 25°C on standard food. Canton S was used as the wild-type strain, and *w; Fb-GAL4* was a gift from Dr. Kuhnlein. All RNAi strains used in this study were obtained from Bloomington Drosophila Stock Center and Vienna Drosophila RNAi Center. Two RNAi lines for Rpd3, *HDAC1*^*JF01401*^and *HDAC1*^*GL01005*^, were targeted to the different *Rpd3* gene regions 3L: 4,628,001 to 4,628,482 and 3L: 4629359 to 4629379, respectively.

### Starvation Assay

Starvation Assays were performed as described previously [39]. For starvation treatments in adults, 3 to 5 day old flies were used. These flies were transferred into vials with 1ml PBS, and the number of living flies were monitored every 3 h. 140 male flies of FB > *Rpd3*RNAi (*w*; *Fb-GAL4* /+; *HDAC1*^*JF01401*^/+), 103 male flies of FB > *kdm2*RNAi (*w*; *Fb-GAL4* / +; *Kdm2*^*HMS00574*^ /+), 132 male flies of FB > *Tip60*RNAi (*w*; *Fb-GAL4* / +; *Tip60*^*GL00130*^ /+), 102 male flies of control (*w*; *Fb-GAL4* / +), 120 male flies of FB > *Rpd3*RNAi (*w*; *Fb-GAL4* /+; *HDAC1*^*JF01401*^/+), 145 male flies of FB > *kdm2*RNAi (*w*; *Fb-GAL4* / +; *Kdm2*^*HMS00574*^ /+), 95 male flies of FB > *Tip60*RNAi (*w*; *Fb-GAL4* / +; *Tip60*^*GL00130*^ /+), 100 male flies of control (*w*; *Fb-GAL4* / +), 183 male flies of FB-GAL4>UAS-*Rpd3*^*JF01401*^ IR (*w*; *Fb-GAL4* /+; *HDAC1*^*JF01401*^/+), 75 male flies of FB-GAL4>UAS-*Rpd3*^*GL01005*^ IR (*w*; *Fb-GAL4* / *HDAC1*^*GL01005*^), 65 male flies of FB-GAL4>UAS-*Rpd3* (*w; Fb-GAL4 / HDAC1*^*Scer/UAS*:*SV5/V5*^), and control (*w*; *Fb-GAL4* / +) were scored. Control animals were always analyzed in parallel under each experimental condition.

### Immunostaining of fat bodies

Three-day old adult flies were collected and dissected in PBS to collect fat bodies, and the fat bodies were fixed in 4% paraformaldehyde for 1 h. After washing several times with PBS containing 0.3% Triton X-100 (PBST) for 10 min each, the samples were treated with 0.01 mg/ml BSA in PBST for 1 h at 4°C and then incubated with the primary antibodies such as anti-Rpd3 IgG (Santa Cruz Biology), anti-Fibrillarin IgG (Abcam), anti-H3K9ac IgG, anti-H3K27ac IgG, anti-H4K8ac IgG, and anti-H4K16ac IgG [40] for 16 h at 4°C. After washing several times with PBST, the samples were incubated for 1 h at 25°C with Alexa Fluor 594-conjugated rabbit anti-goat IgG, Alexa Fluor 488 conjugated goat anti-mouse IgG, or Alexa Fluor 594-conjugated goat anti-mouse IgG. Preparations were examined using confocal laser scanning microscopy (Olympus FV10i). The obtained images were analyzed with MetaMorph (Molecular Devices). All staining intensity measurements are presented as pixel intensities. *Drosophila* fat bodies showed autofluorescence, and this background signal was subtracted from each measured value.

### Western immunoblot analysis

Protein extracts from fat bodies of *Drosophila* adult Canton S flies or flies carrying *w; Fb-GAL4 /+; Rpd3*^*JF01401*^*/+* were prepared. Briefly, the fat bodies were excised from newly enclosed adult male flies and homogenized in a sample buffer containing 50 mM Tris-HCl (pH 6.8), 2% sodium dodecyl sulfate (SDS), 10% glycerol, 0.1% bromophenol blue, and 1.2% β-mercaptoethanol. The homogenates were boiled at 95°C for 5 min and then centrifuged. The supernatants (extracts) were electrophoretically separated on SDS-polyacrylamide gels containing 8% acrylamide and then transferred to polyvinylidene difluoride (PVDF) membranes (Bio-Rad). The blotted membranes were blocked with TBS containing 0.05% Tween 20 and 1% blocking solution (GE healthcare) for 1 h at 25°C, followed by incubation with rabbit anti-Rpd3 IgG (Santa Cruz Biology) at a 1:1,000 dilution for 16 h at 4°C. After washing, the membranes were incubated with goat anti-rabbit IgG (GE healthcare) at a 1:10,000 dilution for 1 h at 25°C. Antibody binding was detected using ECL Western blotting detection reagents (GE healthcare), and images were analyzed with a AE-9300H Ez-Capture MG (ATTO). To ensure equal protein loading in each lane, the membranes were also probed with anti-α-tubulin antibody after stripping the complex of anti-Rpd3 antibody and HRP-conjugated anti-rabbit IgG. For the detection of α-tubulin, mouse anti-α-tubulin monoclonal antibody (1:8,000 dilution, Sigma,) and HRP-conjugated anti-mouse IgG (1:10,000 dilution, GE healthcare) were used as the primary and secondary antibodies, respectively.

### RT-qPCR

Three replicates of 20 starved adult male flies were collected at each starvation period and stored at -80°C. The starvation conditions were the same as those written in the starvation assay method. The collected flies were homogenized with the glass homogenizer on ice. Total RNA was extracted with Trizol ® reagent (Invitrogen) from the whole bodies and synthesized into cDNA using PrimeScript RT reagent kits (TaKaRa), according to the manufacturer’s instructions. Samples were run in duplicates with SYBR^®^ Premix Ex Taq^TM^ II (TaKaRa) using CFX96 touch^TM^ (Biorad), and the data were analyzed with a standard curve-based method calculated with CFX Manager^TM^ software. Specificity of primers was tested with melt curves created by CFX Manager^TM^ software, and agarose gel electrophoresis of amplified fragments. β-tubulin and G6pd were used as an internal control. All *Drosophila* genomic region sequences were based on the FlyBase database (version FB2015_05). Primer sequences are listed below.

18srRNA-F 5’-GGTCTGTGATGCCCTTAGATG-3’,

18srRNA-R 5’-GGACCTCTCGGTCTAGGAAATA-3’,

Rpd3-F 5’-CTGCTCAACTATGGGCTCTATC-3’,

Rpd3-R 5’-CTCATCGGCAGTGGCTTTAT-3’,

Atg9-F 5’-GTTTAAGGCTGGCTACCTACTC-3’,

Atg9-R 5’-AGAAACGCACCAGCTCTATG-3’,

β-tubulin-F 5’-GAGACCTACTGCATCGACAAC-3’,

β-tubulin-R 5’- CAGGGAGACAAGATGGTTCAG-3’

Tip60-F 5’-CTAACCGAGGGATGTCGTTTAC-3’,

Tip60-R 5’-ACTCCTTGATGCTCACAATCTC-3’,

G6pd-F 5’-GAACAAGAACAAGGCCAACC-3’,

G6pd-R 5’-AGGCTTCTCGATAATCACGC-3’

#### ChIP-qPCR

Forty starved male flies were collected at each starvation period and quickly frozen in liquid nitrogen, homogenized, and immediately cross-linked with paraformaldehyde, as described previously [41]. After cross-linking, we performed ChIP using the ChIP Assay Kit (Millipore), according to the manufacturer’s instructions. In brief, cross-linked DNA was fragmented by sonication (Bioruptor, Cosmo Bio) and immunoprecipitated with 0.2μg of anti-Rpd3 IgG, anti-H3K9acIgG, anti-H4K8acIgG, anti-H4K16ac IgG, or control normal mouse IgG for 16 h at 4°C. After washing and reverse cross-linking, DNA was isolated, and RT-qPCR was performed with SYBR Green Master using CFX96 Touch™ (Bio-Rad). The data were analyzed with a standard curve-based method. rRNA promoter sequence refers to the reference [42]. Primer sequences are listed in below.

rRNA promoter region 1-F 5’-ACCTGCCTGTAAAGTTGGATTA-3’,

rRNA promoter region 1-R 5’-AACCGAGCGCACATGATAA-3’

rRNA promoter region 2-F 5’-CAATATGAGAGGTCGGCAAC-3’,

rRNA promoter region 2-R 5’-TATTATCCGCGGAGCCAAGT-3’

rRNA promoter region 3-F 5’-CGACCTCGCATTGTTCGAAAT-3’,

rRNA promoter region 3-R 5’-TATTATCCGCGGAGCCAAGT-3’

rRNA promoter region 4-F 5’-GAGAAACGGCTACCACATCTAA-3’,

rRNA promoter region 4-R 5’-CCTCGGATATGAGTCCTGTATTG-3’

rRNA promoter region 5-F 5’-GGTCTGTGATGCCCTTAGATG-3’,

rRNA promoter region 5-R 5’-GGACCTCTCGGTCTAGGAAATA-3’

rRNA promoter region 6-F 5’-GCTCATGGGTCGATGAAGAA-3’,

rRNA promoter region 6-R 5’-ACAGCATGGACTGCGATATG-3’

rRNA promoter region 7-F 5’-AAGAGTCGTGTTGCTTGATAGT-3’,

rRNA promoter region 7-R 5’-CTTTCCCTCACGGTACTTGTT-3’

Atg9 promoter region 1-F 5’-GAGGGCCAAGGTTCGTATTTA-3’,

Atg9 promoter region 1-R 5’-TGCCCGTTTCCCAAATAGAG-3’

Atg9 promoter region 2-F 5’-CTGGCTGGATTCTTTCTCTCTT-3’,

Atg9 promoter region 2-R 5’-AGTACGACATCTCGACCTTCTA-3’

#### Polysome profile analysis

One hundred sixty starved male flies were collected at each starvation period, quickly frozen in liquid nitrogen, homogenized with a mortar in liquid nitrogen and then polysome buffer was added, as described before [43]. Preparation of the extracts and sucrose gradient separation for the polysome analysis were carried out using a gradient master 107–201M and fractionator 152–002 (BioComp Instruments) by the methods of Inada and Aiba [44]. The percentage of polysomal ribosomes was determined by the method of Hofmann *et al*. [45].

#### Statistical analysis

Unpaired two-tailed Student t-tests were performed to analyze the results of the immunostaining analysis and RT-qPCR. Log-rank tests were performed to analyze the results of the viability assays using GraphPad Prism 6 software, which uses the Kaplan-Meier estimator to calculate survival fractions, as well as median and maximum survival values. Compared results were considered statistically significant when the *P-value < 0.05 and ** P-value < 0.01.

## Supporting Information

S1 Fig Viability of the *Rpd3* knockdown and *Rpd3* overexpressing flies under starvation stress. Percent survival of adult male flies at indicated hours starved are shown. FB-GAL4>UAS-*Rpd3*^*JF01401*^ IR (*w*; *Fb-GAL4* /+; *HDAC1*^*JF01401*^/+), FB-GAL4>UAS-*Rpd3*^*GL01005*^ IR (*w*; *Fb-GAL4* / *HDAC1*^*GL01005*^), FB-GAL4>UAS-*Rpd3* (*w; Fb-GAL4 / HDAC1*^*Scer/UAS*:*SV5/V5*^), control (*w*; *Fb-GAL4* / +).
(TIF)

S2 Fig *Rpd3* and *Tip60* knockdown efficiency in fat bodies of FB-GAL4>UAS-*Rpd3*^*JF01401*^IR and FB-GAL4> UAS-*Tip60*^*GL00130*^IR flies. **A**. Western immunoblot analysis. Fat bodies from adult flies, Canton S (WT) or *Rpd3* knockdown flies, FB> *Rpd3*RNAi (*w*; *Fb-GAL4* /+; *HDAC1*^*JF01401*^/+) starved for 3 h and 6 h were analyzed by Western blot using anti-Rpd3 IgG. Two bands were detected (arrowheads). The upper band corresponds to 93.8kDa, and the lower band corresponds to 67.7kDa. The α-tubulin was used as a loading control. **B**. Relative intensities of the lower band of Rpd3 normalized to α-tubulin are shown for wild type (WT) and *Rpd3* knockdown (FB> *Rpd3*RNAi) flies at 3 h and 6 h starvation. **C.** Relative intensities of the upper band of Rpd3 normalized to α-tubulin are shown for wild type (WT) and *Rpd3* knockdown (FB> *Rpd3*RNAi) flies at 3 h and 6 h starvation. **D.** Relative *Tip60* mRNA expression levels in knockdown flies. Fat bodies from adult Canton S (WT) or *Tip60* knockdown flies, FB>*Tip60*RNAi (*w*; *Fb-GAL4* / +; *Tip60*^*GL00130*^ /+) were analyzed by RT-qPCR. *Tip60* mRNA expressions were normalized with levels of *G6pd* mRNA. ** P-value < 0.01, n = 3.
(TIFF)
