# Supplementary figures and images for: The Histone Deacetylase Gene Rpd3 Is Required for Starvation Stress Resistance
Source: PLoS One. 2016 Dec 1;11(12):e0167554. doi: 10.1371/journal.pone.0167554 (PMC5132236; doi:10.1371/journal.pone.0167554)

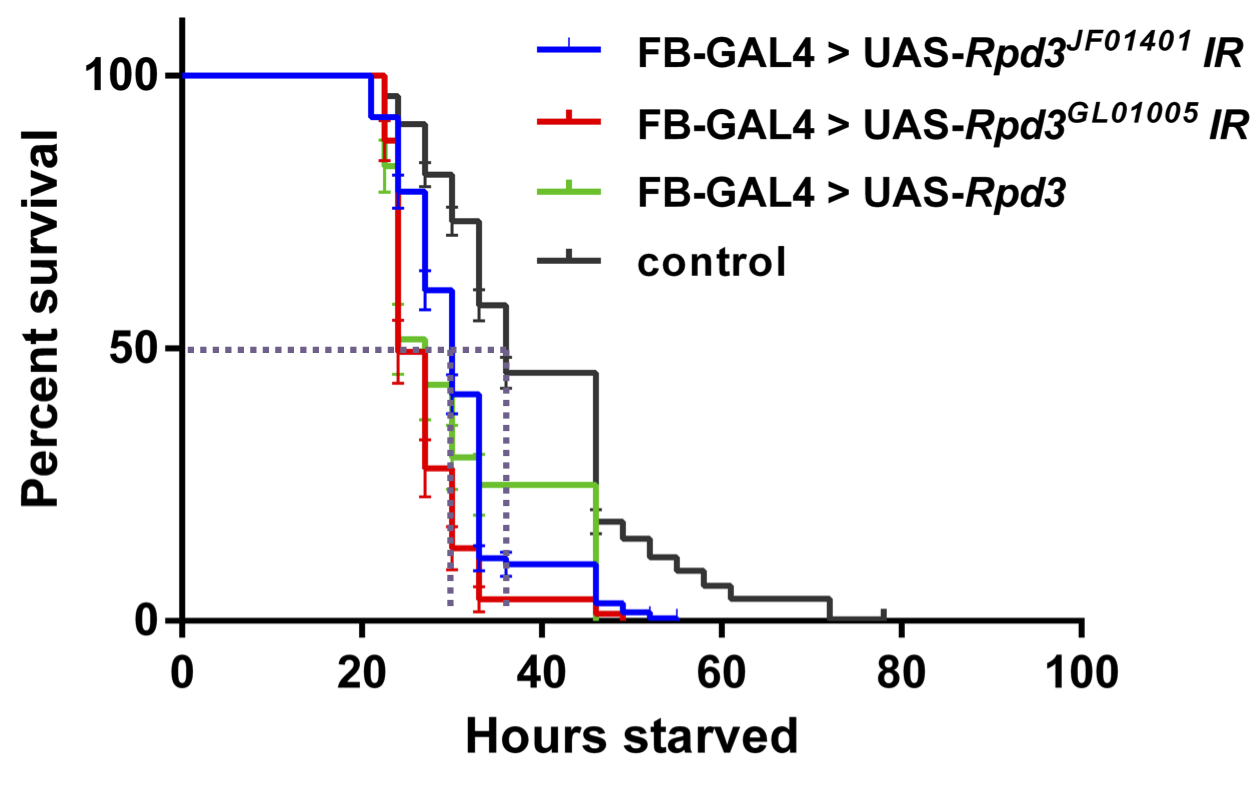

Supplement: S1 Fig — Percent survival of adult male flies at indicated hours starved are shown. FB-GAL4>UAS-Rpd3JF01401 IR (w; Fb-GAL4 /+; HDAC1JF01401/+), FB-GAL4>UAS-Rpd3GL01005 IR (w; Fb-GAL4 / HDAC1GL01005), FB-GAL4>UAS-Rpd3 (w; Fb-GAL4 / HDAC1Scer/UAS:SV5/V5), control (w; Fb-GAL4 / +). (TIF) [file pone.0167554.s001.tif]

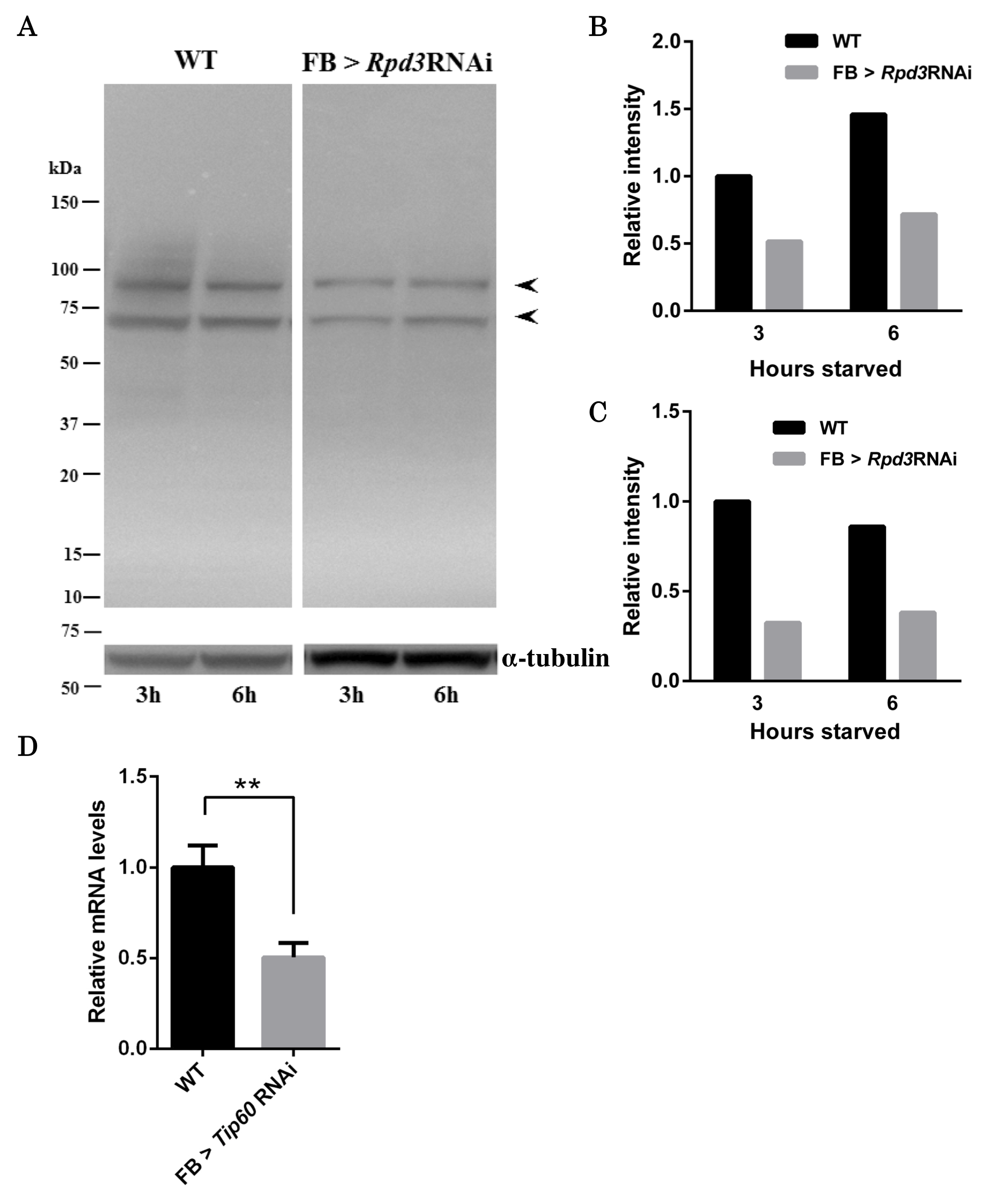

Supplement: S2 Fig — A. Western immunoblot analysis. Fat bodies from adult flies, Canton S (WT) or Rpd3 knockdown flies, FB> Rpd3RNAi (w; Fb-GAL4 /+; HDAC1JF01401/+) starved for 3 h and 6 h were analyzed by Western blot using anti-Rpd3 IgG. Two bands were detected (arrowheads). The upper band corresponds to 93.8kDa, and the lower band corresponds to 67.7kDa. The α-tubulin was used as a loading control. B. Relative intensities of the lower band of Rpd3 normalized to α-tubulin are shown for wild type (WT) and Rpd3 knockdown (FB> Rpd3RNAi) flies at 3 h and 6 h starvation. C. Relative intensities of the upper band of Rpd3 normalized to α-tubulin are shown for wild type (WT) and Rpd3 knockdown (FB> Rpd3RNAi) flies at 3 h and 6 h starvation. D. Relative Tip60 mRNA expression levels in knockdown flies. Fat bodies from adult Canton S (WT) or Tip60 knockdown flies, FB>Tip60RNAi (w; Fb-GAL4 / +; Tip60GL00130 /+) were analyzed by RT-qPCR. Tip60 mRNA expressions were normalized with levels of G6pd mRNA. ** P-value < 0.01, n = 3. (TIFF) [file pone.0167554.s002.tiff]
